# Utilization of a genetically modified muscle flap for local BMP-2 production and its effects on bone healing: a histomorphometric and radiological study in a rat model

**DOI:** 10.1186/s13018-015-0196-6

**Published:** 2015-04-29

**Authors:** Florian M Lampert, Arash Momeni, Filip Filev, Nestor Torio-Padron, Günter Finkenzeller, G Björn Stark, Dominik Steiner, Georgios Koulaxouzidis

**Affiliations:** Department of Plastic and Hand Surgery, University of Freiburg Medical Center, Hugstetterstr. 55, D-79106 Freiburg, Germany; Division of Plastic and Reconstructive Surgery, Stanford University Medical Center, 770 Welch Road, Suite 400, Palo Alto, CA 94304-5715 USA; Department of Ophthalmology, University MedicalCenter Hamburg-Eppendorf, Haus West 40 (W40), Martinistr. 52, D-20246 Hamburg, Germany; Department of Plastic and Hand Surgery, University Hospital of Erlangen, Friedrich-Alexander-University of Erlangen-Nürnberg, Krankenhausstrasse 12, 91054 Erlangen, Germany

**Keywords:** Bone regeneration, Gene therapy, Reconstructive microsurgery, Bone morphogenetic protein, BMP-2, Regenerative medicine, Translational medicine, Tissue engineering

## Abstract

**Aim of the study:**

We developed an experimental rat model to explore the possibility of enhancing the healing of critical-size bone defects. The aim of this study was to demonstrate the feasibility of this concept by achieving high local BMP-2 expression via a transduced muscle flap that would facilitate bony union while minimizing systemic sequelae.

**Methods:**

The transduction potential of the adenoviral vector encoding for BMP-2 was tested in different cell lines *in vitro. In vivo* experiments consisted of harvesting a pedicled quadriceps femoris muscle flap with subsequent creation of a critical-size defect in the left femur in Sprague-Dawley rats. Next, the pedicled muscle flap was perfused with high titers of Ad.BMP-2 and Ad.GFP virus, respectively. Twelve animals were divided into three groups comparing the effects of Ad.BMP-2 transduction to Ad.GFP and placebo. Bone healing was monitored radiologically with subsequent histological analysis post-mortem.

**Results:**

The feasibility of this concept was demonstrated by successful transduction *in vitro* and *in vivo* as evidenced by a marked increase of BMP-2 expression. The three examined groups only showed minor difference regarding bone regeneration; however, one complete bridging of the defect was observed in the Ad.BMP-2 group. No evidence of systemic viral contamination was noted.

**Conclusions:**

A marked increase of local BMP-2 expression (without untoward systemic sequelae) was detected. However, bone healing was not found to be significantly enhanced, possibly due to the small sample size of the study.

## Introduction

A critical-size bone defect is, by definition, unable to regain bony union without intervention. The gold standard treatment of such defects is transplantation of autologous bone [1]. However, donor-site morbidity and limited availability represent drawbacks of this approach. In contrast, allogenic bone grafts are readily available; however, they have a considerably lower osteoinductive potential and bear the risk of transplant rejection as well as transmission of infectious diseases [2]. Bone regeneration through topical application of synthetic growth factors has already been demonstrated. In particular, administration of BMP-2 has been used with considerable success in orthopedic surgery and neurosurgery [3-6]. Limitations of this approach, however, include 1) high cost and 2) need for repeated applications secondary to the short half-life of the protein. Other experimental therapies include the development of injectable biological bone substitutes as well as exposure of human mesenchymal stem cells to BMP-2 *ex vivo*, thus, inducing their osteoblastic differentiation [7,8]. Transduction of target cells with a replication-deficient adenovirus, designed to carry cDNA encoding for BMP-2, represents a novel approach for local delivery of BMP-2 to the region of interest. Previous studies have shown significant improvement of bone regeneration after local application of BMP-2 carrying adenoviruses to the bone defect in a rat model [9-11]. Injecting very high virus titers directly into the bone defect, however, can lead to systemic spreading of the virus, with a resultant negative impact on other organs, along with initiation of potentially severe or even lethal immunological reactions [12]. In order to minimize the risk of systemic contamination of the replication-deficient adenovirus used in the present study, a pedicled quadriceps muscle flap was harvested and selectively infused with the virus. The concept of using genetically altered soft tissue flaps as biologic pumps has been demonstrated by Liu et al. and Michaels et al. in a rat model [13-15]. The present study represents the first attempt of assessing whether muscle flaps can be altered genetically so as to not only provide soft tissue coverage but also enhance osseous healing by local production of growth factors, as muscle tissue is known to possess an ample quantity of mesenchymal stem cells capable of osteoblastic differentiation [8]. Furthermore, BMP-2 has an angiogenetic potential, which could aid in facilitating the healing process [16].

## Materials and methods

### Viral vector

Replication-deficient viruses were used in the present study, i.e., Ad.BMP-2 and Ad.GFP vector (courtesy of Dr. O. Betz, Munich). These vectors represent two subtypes of a replication-deficient E1/E3-deleted adenovirus type 5 vector driven by a cytomegalovirus promoter. Human BMP-2 cDNA and GFP (green fluorescent protein) cDNA were cloned into the E1 domain, respectively. The virus was amplified in a HEK 293 cell line and titered using HEK 293 standard plaque assay. The concentration of infective particles is indicated as plaque-forming units per milliliter (PFU/ml) and set in relation to the number of target cells as MOI (multiplicity of infection). Adenoviral vector efficiency was first assessed *in vitro* in the following human and rat cell types:

- MSC - human mesenchymal stem cells (obtained by bone marrow biopsy at the University of Freiburg Medical Center)
- Cal-72 human osteosarcoma cells (ACC 439 Deutsche Sammlung von Mikroorganismen und Zellkulturen Braunschweig, Germany)
- SaOs-2 human osteosarcoma cells (ACC 243 Deutsche Sammlung von Mikroorganismen und Zellkulturen Braunschweig, Germany)
- HUVEC - human umbilical vein endothelial cells (Promocell, Cat. No. C-12250, Heidelberg, Germany)
- Sprague Dawley rat myocyte cell culture isolated from the quadriceps muscle

The cultured cells were incubated on six-well plates at 37°C/5% CO_2_ until confluence was reached, then 1 ml of virus suspension (Ad.GFP or Ad.BMP-2) was added to the medium. Five days after transduction, the efficiency of transduction was evaluated. BMP-2 levels in the culture medium were measured with a human BMP-2-specific ELISA assay (DBP 200). The cultures transfected with GFP-2 were examined microscopically for UV fluorescence.

### Animals

Adult male Sprague Dawley rats weighing 400–450 g (Charles River, Sulzfeld, Germany) were used for *in vivo* experiments. All animals were kept in an approved animal care facility with 12-h light/dark cycles and were allowed food and water *ad libitum* and unrestricted activity after surgery. All animal studies were approved by the ethical review board of Baden-Württemberg (G 07/33) and conducted in compliance with the guidelines specified in German legislation concerning animal experiments (Tierschutzgesetz, §§ 7 to 9).

Animals were divided into three groups:

- Group 1: BMP group (*n* = 4): muscle flap infused with 1-ml Ad.BMP-2 suspension (8 × 10^9^ PFU/ml),
- Group 2: GFP group (*n* = 4): muscle flap infused with 1-ml Ad.GFP suspension (6 × 10^9^ PFU/ml),
- Group 3: control group (*n* = 4): muscle flap infused with 1 ml of sterile normal saline.

### Surgical procedure

**Figure 1:**
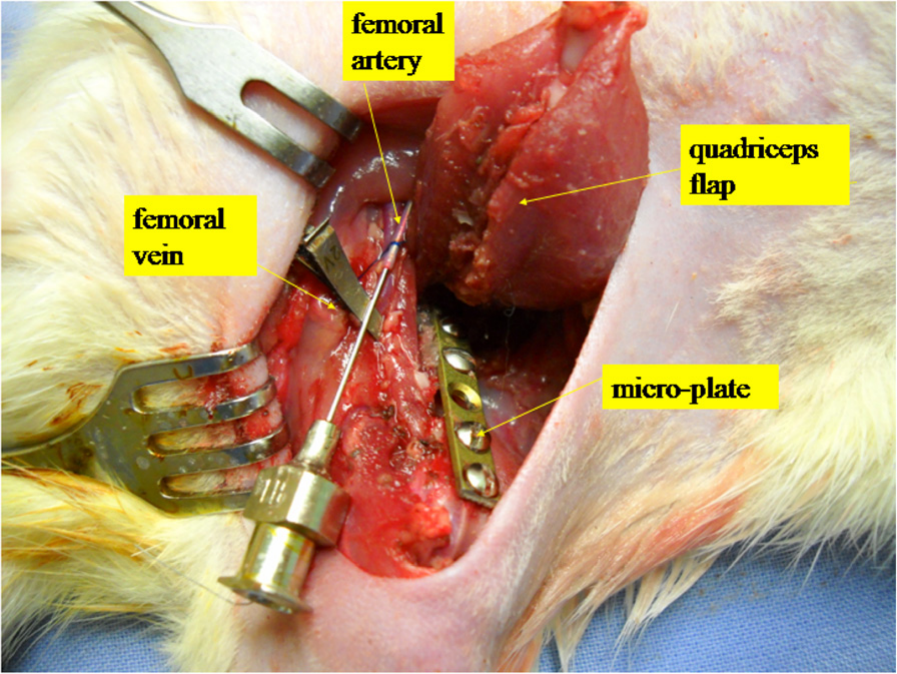
Surgical site. The image demonstrates the elevated quadriceps muscle flap only attached by its pedicle with the Pravaz cannula in the afferent artery. The efferent vein is clamped and the microplate is displayed bridging the femoral critical-size defect. Via the Pravaz cannula, 1.0 ml of virus solution (8 × 10^9^ plaque-forming units (BMP group) or 6 × 10^9^ plaque-forming units (GFP group)) were injected (incubation time 1 h). A micro-plate (KLSMartin, Tuttlingen, Germany) secures the femur after performing a critical-size defect (5 mm) in the middle of the femoral shaft.

Surgery was performed under general anesthesia using isoflurane (Forene®, Abbott, Switzerland) at a flow rate of 1.5 l/min. The left lower extremity was clipped and prepped using PVP-iodine solution (Beta-Isodona, Mundipharma, Limburg/Lahn, Germany). A 3-cm skin incision was made on the medial aspect of the left thigh with subsequent dissection through the subcutaneous tissue until the quadriceps muscle was visualized. The muscle was carefully harvested and left attached only via its pedicle. All vascular dissections were performed under the microscope. Next, blood flow was interrupted by clamping the femoral vessels distal to the iliac ligament. The femoral artery and vein were then transected distal to the knee joint and a blunt 0.2-mm Pravaz cannula was inserted into the artery, while an 8-mm microsurgical vascular clamp (Roboz Surgical Instrument Co., Inc. RS-6470) was placed on the vein. Then, 1 ml of virus solution containing 8 × 10^9^ plaque-forming units (BMP group) or 6 × 10^9^ plaque-forming units (GFP group) was injected into the artery (Figure 1), followed by an incubation period of 1 h. Controls were treated by injecting 1 ml of sterile normal saline. The femur defect was created during the incubation period. A micro-plate (KLSMartin, Tuttlingen, Germany) was secured to the femur using four screws [17]. After fixation, a 5-mm defect (i.e., critical-size defect) [10] was created in the middle of the femoral shaft using a burr. After the incubation period, the venous clamp was removed and the flap was flushed with sterile normal saline solution (0.9%) via the femoral artery to remove any non-incorporated viruses. The surgical site was irrigated with PVP-iodine solution and saline to minimize local viral contamination [18]. After insetting the flap into its original location using 5-0 Vicryl® (Ethicon, Germany), skin closure was performed with Prolene® 4-0 (Ethicon, Germany). All animals were given prophylactic antibiotics (Borgal® Sulfadoxinum 200 mg, Trimethoprimum 40 mg, 15 mg/kg). Tamgesic® (15 μg) was administered subcutaneously for pain control for 5 days postoperatively.

**Figure 2:**
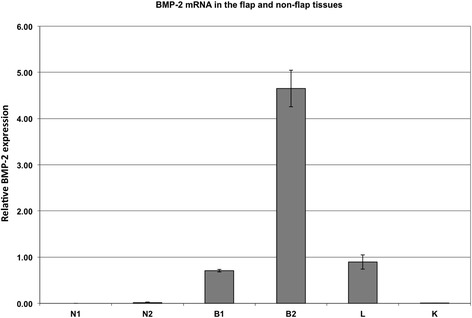
Quantification of transduction efficacy (RT-PCR). To test the transduction efficiency *in vivo*, Ad.BMP was injected into the quadriceps muscle of two animals. Quantitative real-time RT-PCR was performed comparing the animals from the BMP group 3 days after transduction (B1, B2) to untreated animals (N1, N2). To exclude systemic effects, the contralateral quadriceps of the treated animals was analyzed (K). Furthermore, we sacrificed one animal after muscle flap perfusion (L). Both flaps (B1, B2) showed increased BMP-2 levels compared to the contralateral quadriceps (K) or the flap of control animals (N1, N2).

To directly assess the transduction efficiency *in vivo*, 1 ml Ad.BMP or Ad.GFP virus solution was intraoperatively injected in multiple layers into the quadriceps muscles of three (2× Ad. BMP, 1× Ad.GFP) additional animals. Transduction efficacy was examined by quantitative real-time RT-PCR and UV fluorescence, respectively (Figure 2).

### BMP-2 ELISA

We used a BMP-2 ELISA kit (R&D Systems, DBP-200 Quantine) to quantify BMP-2 expression in both *in vitro* and *in vivo* experiments analyzing cell culture supernatants and tissue sample lysates, respectively. The samples were normalized to equal protein quantities, either 100 or 300 μg in different experiments, using a BCA protein Assay Reagent Kit® (Pierce, Thermo Scientific) and added to each well of the ELISA kit. Absorbance was measured at 450 nm and correlated to a standard curve obtained by measuring pre-equilibrated standard samples. The mean minimum detectable dose was 11 pg/ml. All test samples were examined in duplicates or triplicates.

### Quantitative real-time RT-PCR

TaqMan RT-PCR was carried out as previously described [19]. Total RNA was isolated from tissue samples using the TRIzol method [20]. Total RNA (0.5 μg) was treated with 3 units of deoxyribonuclease I (DNase I, Invitrogen, Karlsruhe, Germany) to digest genomic DNA contamination. Random-primed cDNA synthesis was performed using 0.5 μg of DNase I-treated total RNA and 50 units of AffinityScript reverse transcriptase according to the manufacturer’s instructions (Stratagene, La Jolla, USA). TaqMan PCR assays were performed in 384-well optical plates on a LightCycler (Roche, Mannheim, Germany) using Absolute QPCR ROX Mix (Abgene, Hamburg, Germany) according to the manufacturer’s instructions. The thermal cycling conditions were 95°C for 15 min followed by 50 cycles at 95°C for 15 s and at 60°C for 1 min. Oligonucleotide primers and probes for human GAPDH (GADPH forward: 5′-TGGGCTACACTGAGCACCAG-3′; GAPDH reverse: 5′-CAGCGTCAAAGGTGGAGGAG-3′, GAPDH probe: 5′-FAM- TCTCCTCTGACTTCAACAGCGACACCC-TAMRA-3′) were designed using Primer Express (Applied Biosystems, Foster City, USA) according to company guidelines. Oligonucleotide primers and TaqMan probe for human BMP-2 (Cat. Nr. Hs00154192) were purchased from Applied Biosystems (Foster City, CA, USA). Assays were performed in triplicate. Data were analyzed using the relative standard curve method, with each sample being normalized to the housekeeping gene GAPDH.

### Determining systemic spread of the viral vector

Samples from the spleen, liver, and lung from all animals in the Ad.GFP group were cut into 10-μm cryo-sections and examined using fluorescence microscopy.

### Radiographic evaluation

**Figure 3:**
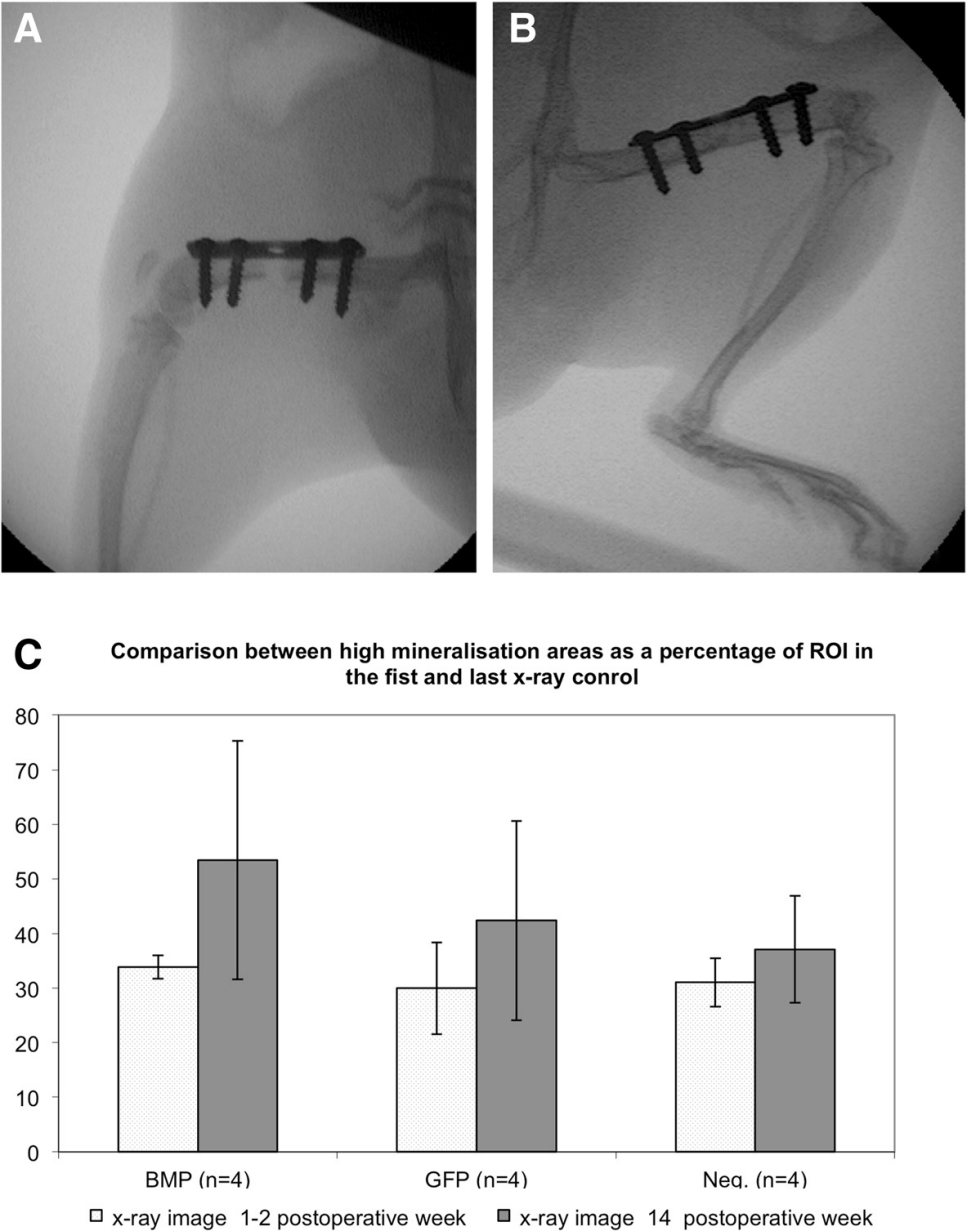
Radiographic evaluation of bone formation. Animals were positioned prone with the left hind limb externally rotated for x-ray imaging. X-ray images were obtained between the third and thirteenth day after surgery **(A)** and on the day of euthanasia (14 weeks postoperatively **(B)**). Here, we present images from an animal of the Ad.BMP-2 group. The images were analyzed assessing radiological density by gray-scale analysis. The region of interest used was defined as the area between the second screw and third screw (distally). An increase of high-density tissue, corresponding to mineralized bone between the first and last x-ray was seen in all three groups **(C)**. The increase was highest in the Ad.BMP-2 group.

Prior to x-ray imaging, animals were anesthetized by intramuscular administration of ketamine (10 mg per 100 g of body weight) and xylazine (0.25 mg per 100 g of body weight). Animals were then positioned prone with the left hind limb externally rotated. An x-ray image was obtained between the third and thirteenth day after surgery and on the day of euthanasia (14 weeks postoperatively) using an OEC Mini 6600 C-arm x-ray-unit (GE OEC Medical Systems GmbH 90530 Wendelstein) (Figure 3A). Radiological density was determined by gray-scale analysis using GIMP (GIMP, GNU General Public License) image software [21]. The region of interest used in the radiographic evaluation was defined as the area between the second screw (proximally) and third screw (distally), containing the whole cortex-to-cortex thickness of the femur.

### Histological study

**Figure 4:**
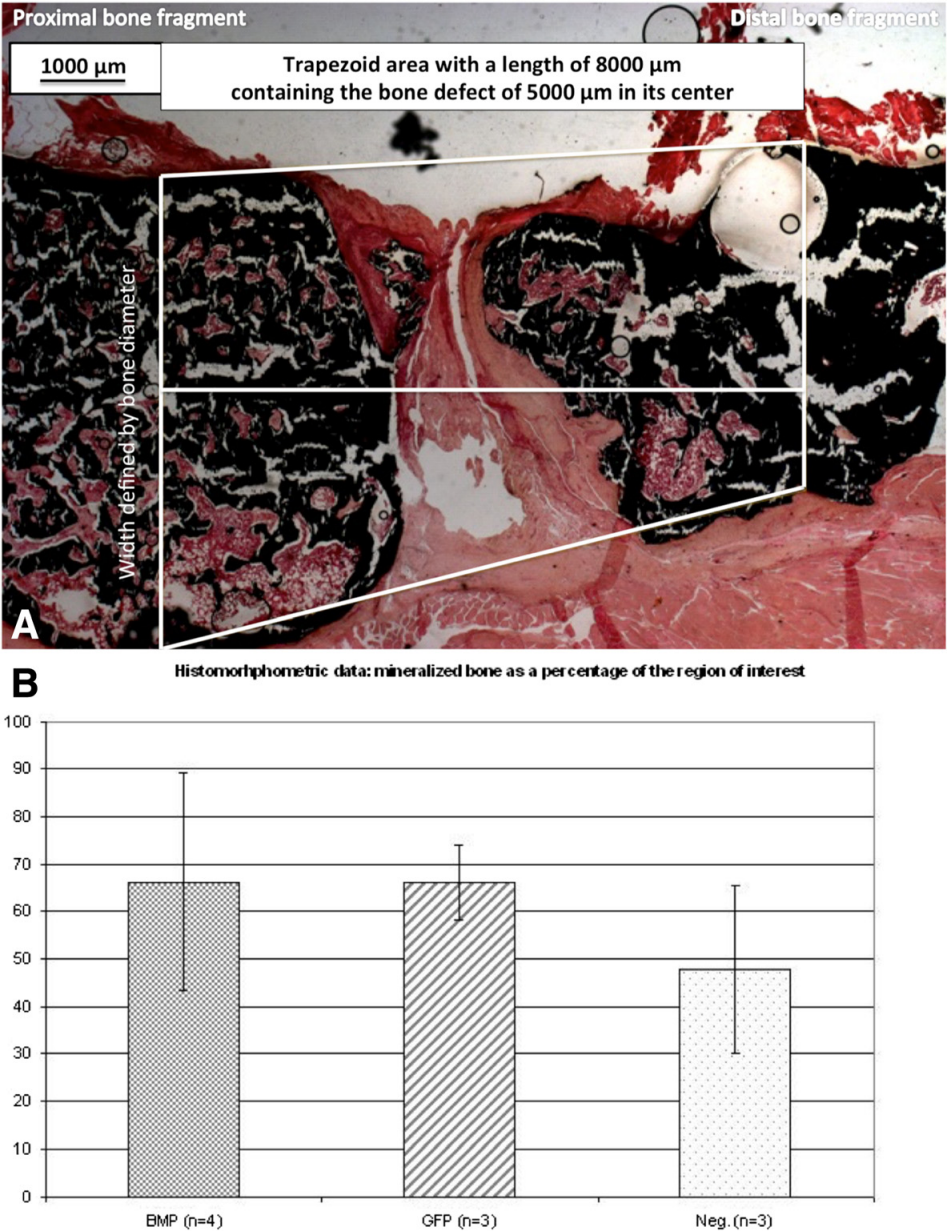
Histomorphometric analysis of bone formation. For histomorphologic analysis of the mineralized area, Von Kossa stainings of samples of the left femur were performed. Histomorphometric analysis of the mineralized area was performed within a region of interest, defined as a trapezoid area with a length of 8,000 μm containing the defect in its center. The parallel sides of this trapezoid were perpendicular to the axis of the femur shaft and stretch between the cortical bone of the proximal and distal bone fragment. The defect area was marked manually and then compared to the total area of the region of interest (**(A)** 25×). Although nonunions were seen in the majority of animals, pronounced inter-individual differences within the groups were detected regarding bone formation. However, in one case (Ad.BMP-2 group), a complete union was achieved. A statistical comparison of the three groups showed little difference regarding the area of mineralized bone compared to the entire region of interest **(B)**.

Euthanasia was performed by CO_2_ asphyxiation after the animals have been put under general anesthesia using isoflurane (Forene® Abbott, Switzerland) at a flow rate of 1.5 l/min. After euthanasia, the left femur was removed and placed in 4% formaldehyde for 24 h. For histomorphometric analysis, samples were embedded into methyl-methacrylate-based hard plastic, without prior demineralization of the bone using the Technovit 9100 kit (Heraeus Kulzer GmbH & Co.) and cut into 8-μm sections. Von Kossa standard staining was used for all sections. Images were obtained (Zeiss Axiocam, Axiovert) and analyzed using GIMP image software. Three sections showing the greatest cortex-to-cortex thickness were evaluated. A region of interest was defined as a trapezoid area with a length of 8,000 μm containing the defect in its center. The parallel sides of this trapezoid were perpendicular to the axis of the femur shaft and stretch between the cortical bone of the proximal and distal bone fragment (Figure 4A). The defect area was marked manually and then compared to the total area of the region of interest.

### Statistical analysis

All data are presented as mean ± standard error of the mean. Due to the small sample size, no further statistical analysis was performed.

## Results

### In vitro

**Figure 5:**
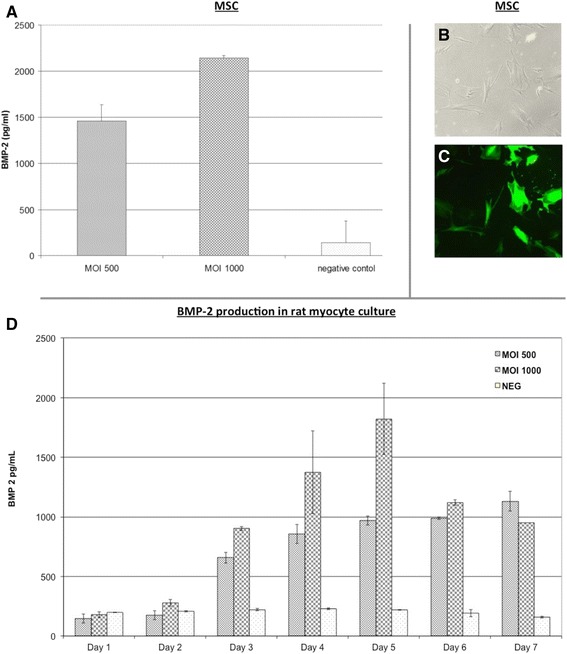
*In vitro* quantification of transduction efficacy. When determining the efficacy of transduction in human mesenchymal stem cells, we observed an overwhelming (10- to 24-fold) increase of BMP-2 in cell culture supernatants in transfected cells compared to controls. BMP-2 expression increased after the multiplicity of infection (MOI) was doubled from 500 to 1,000 PFU/cell **(A)**. *In vivo* microscopy of cell cultures 5 days after transduction with Ad.GFP in native (**(B)** 100×) and UV light (**(C)** 100×) highlights a successful transduction of human mesenchymal stem cells. A peak in BMP-2 production on the fifth day after transduction was observed when using a MOI of 1,000 PFU/cell and a plateau after the fifth day when MOI 500 was used **(D)**.

When determining the efficacy of transduction in human mesenchymal stem cells (Figure 5A) and the Cal-72 human osteosarcoma cell line (data not shown), we observed an overwhelming (10- to 24-fold) increase of BMP-2 in cell culture supernatants in transfected cells compared to controls. In the MSC group, BMP-2 expression increased after the multiplicity of infection (MOI) was doubled from 500 to 1,000 PFU/cell. Transduction with Ad.GFP was confirmed by UV light microscopic analysis (Figure 5B). Next, we analyzed the kinetics of BMP-2 expression over time in a culture of Sprague-Dawley rat myocytes (Figure 5C). A peak of BMP-2 expression was seen on the fifth day after transduction when using an MOI of 1,000. BMP-2 expression demonstrated a stable plateau after the fifth day with roughly identical values on the sixth an seventh day post-transduction in both MOI 500 and MOI 1,000 cultures.

### In vivo

**Figure 6:**
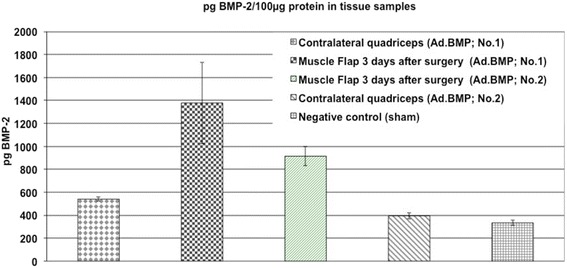
BMP-2 expression in tissue samples. BMP-2 expression in the transfected flap on postoperative day 3 was compared to a sample from the contralateral quadriceps. Both flaps showed increased BMP-2 levels compared to the contralateral quadriceps and the flap of control animals.

To test the transduction efficiency *in vivo*, the Ad.BMP was injected into the quadriceps muscle of two animals as described above. Furthermore, we sacrificed one animal after muscle flap perfusion (executed as described in surgical procedure). Quantitative real-time RT-PCR was performed comparing the animals from the BMP group 3 days after transduction to untreated animals (Figure 2). Furthermore, 1 ml containing 6 × 10^9^ PFUs of Ad.GFP was injected directly into the quadriceps muscle of one animal as described above and showed GPF fluorescence when examined under UV light 3 days after transduction. As shown in Figure 6, we compared Ad.BMP-2 expression in the transfected flap on postoperative day 3 to a sample from the contralateral quadriceps. Both flaps showed increased BMP-2 levels compared to the contralateral quadriceps or the flap of control animals. This finding was consistent with our PCR findings (Figure 2), which demonstrated the presence of human BMP-2 mRNA only in transfected flaps and not in the contralateral quadriceps.

### Radiographic analysis

An increase of high-density tissue, corresponding to mineralized bone between the first and last x-ray, was seen in all three groups. The increase was highest in the Ad.BMP-2 group showing an average of 53% high-density tissue in the region of interest vs. 42% and 37% in the GFP and control group, respectively (Figure 3B).

### Histological evaluation

Although nonunions were seen in the majority of the animals, pronounced inter-individual differences within the groups were detected regarding bone formation. A comparison between the three groups showed little difference regarding the area of mineralized bone compared to the entire region of interest (Figure 4B). One case of mineralized bony union was seen in group 1.

### Systemic spread of the viral vector (i.e., systemic contamination)

Samples from the spleen, liver, and lung from all animals in the Ad.GFP group were cut into 10-μm cryo-sections and microscopically examined under UV light. Fluorescence was not observed in any of the sections.

### Perioperative mortality

In the course of experiments, we lost five animals during the operation in the 24-h period after the operation. Four animals were excluded from the study and underwent euthanasia after a dislocation of the osteosynthesis, two due to wound dehiscence and one due to an ischemia of the operated limb.

## Discussion

Gene therapy is an innovative approach for treatment of large bone defects. Previous studies demonstrated that the adenoviral vector is particularly well equipped for utilization in this regard [9-11]. Its favorable characteristics include easy handling and ability to transduce target tissue effectively. Furthermore, being replication deficient, it does have a favorable safety profile. In order to decrease technical complexity, yet continuing to minimize systemic toxicity and immune response, we modified the concept introduced by Michaels et al. by using the quadriceps femoris muscle as a pedicled flap [14,15]. The current model was chosen, as it resembles the clinical setting, in which muscle flaps are used to cover bone defects. The concept of being able to use a muscle flap not only as soft tissue coverage but also as a biologic pump that secretes growth factors stimulating bone regeneration is intriguing. While a quadriceps femoris free flap has been previously described [22], we employed its modification as a pedicled flap, thus, reducing technical complexity, ischemia time, and overall risk of flap failure. In none of the animals in group 1 did we detect an increase of BMP-2 expression in tissues other than the transduced muscle flap. PCR showed human BMP-2 mRNA in the transduced flap and absence thereof in the contralateral quadriceps. Samples from the spleen, liver, lung, and contralateral quadriceps of animals in group 2 were examined microscopically and did not display any fluorescence, thus confirming indirectly the lack of undesired systemic transduction.

In the present study, we were able to prove the concept that delivery of BMP-2 via a genetically altered muscle flap is possible without any measurable systemic contamination. Unfortunately, a significant difference between the groups with respect to ossification of the critical-size defects could not be observed. An explanation for this observation is the small sample size with resultant limited power in the present study. We achieved an overwhelming increase of BMP-2 expression in human and rat cell types *in vitro*, which remained stable on a high level in the first week after transduction. The time kinetics of BMP-2 expression paralleled reports of transgene expression being highest between the 5th and 14th day post-transduction [15]. The fact that even in untreated cell culture supernatants and tissue samples, the BMP-2 ELISA kit detected BMP-2 levels higher than the minimum detectable concentration suggests some cross-reactivity with rodent proteins. An additional effect could be the induction of endogenous BMP-2 expression as a result of bone injury, i.e., creation of the critical-size femoral defect. This phenomenon has already been shown for a myriad of growth factors including PDGF, FGF, IGF, TGF-β, and VEGF [23-27].

*In vivo* BMP-2 expression was comparatively lower. Human BMP-2 mRNA was however detected in the muscle flaps of all Ad.BMP-2 transduced animals, thus, proving that transduction was successful.

Follow-up x-ray images revealed that the Ad.BMP-2 group showed the most pronounced improvement in the radiographical score for high mineralization. Due to the small sample size and considerable inter-individual differences, however, statistical significance was not achieved. The same applies for the histomorphometric data of the BMP-2 group. In conclusion, we were able to demonstrate that continuous local expression of BMP-2 is possible by means of muscle transduction. Shortcomings of topical BMP-2 application, such as associated cost and necessity for repeated applications secondary to the short half-life of the protein, can, thus, be addressed successfully. Most notably, this approach does not result in systemic contamination with viral particles. Clinical application would be particularly attractive as the need for harvesting autologous bone for reconstruction of composite defects could be reduced or even averted. While this development may not seem to be in reach at present, further development of this model employing larger groups and later larger animal model would provide important clinical data regarding applicability in a clinical setting.

Limitations of the study include the small sample size. The perioperative loss of 12 animals was likely caused by the relatively long, complex, and invasive surgical procedure consisting of microsurgical flap dissection, osteosynthesis, and creation of the critical-size defect as well as the incubation period of 1 h. The loss of animals was evenly spread in all three groups.

Compared to other BMP-2 application models such as direct recombinant BMP injections in the fracture site or the use of biodegradable BMP carrier [28], the use of an adenovirus as a vehicle of gene delivery has the disadvantage of a possible immune response which can lead to a shorter duration of BMP-2 expression [29]. The results of the present study correspond to data published by Willett et al. showing the reduced osteogenic potential of BMP-2 *in vivo* after a composite muscle-bone injury compared to osseous injury alone, making further investigation in this topic desirable [30]. Additional studies need to be carried out with larger sample size to address limitation of the present study. These experiments then need to be followed by large animal studies to confirm the effects seen in the rat model. As stated above, a larger animal model would also be appropriate to reduce the observed high dropout rate, since the grade of invasiveness and duration of the procedure are substantial in a small rodent model. Further modifications could include a transient immunosuppression shortly before and after surgery as proposed by Okubo [31] which may improve the osteogenetic potential of the present model.

## Conclusions

Gene therapy is a promising option for the treatment of critical-size bony defects. We explored the effects of adenovirus-mediated, self-sustained BMP-2 production by a transduced pedicled quadriceps muscle flap in a critical-size femoral defect in a rat model. Our aim was to achieve high local BMP-2 expression while simultaneously minimizing the risks of systemic contamination and initiation of an adverse immune response. While statistical significance was not observed with respect to enhanced bone formation, we were able to demonstrate effective transduction and increased local BMP-2 expression by transduced flaps, both without evidence of systemic viral contamination.
